# Development of disease‐modifying therapies against Alzheimer's disease

**DOI:** 10.1111/pcn.13681

**Published:** 2024-06-06

**Authors:** Takeshi Iwatsubo

**Affiliations:** ^1^ Department of Neuropathology, Graduate School of Medicine The University of Tokyo Bunkyo‐ku Japan; ^2^ National Institute of Neuroscience, National Center of Neurology and Psychiatry Kodaira Japan

**Keywords:** Alzheimer, disease‐modifying therapy

## Abstract

To successfully develop disease‐modifying therapies (DMT) against Alzheimer's disease (AD), it is important to target the mild stage of the disease, before the pathological changes progress and dementia symptoms are fully manifested. To this end, the AD Neuroimaging Initiative (ADNI), a large‐scale observational study, was initiated in the U.S. with the goal of development of DMT that are effective in the early stages of mild cognitive impairment (MCI) by utilizing imaging and biomarkers. In Japan, J‐ADNI enrolled and followed up 537 patients, mainly with MCI, and established a platform for evaluation including amyloid PET, and demonstrated a high similarity in the clinical course of amyloid‐positive MCI (prodromal AD) in Japan and the U.S. In 2023, the anti‐Aβ antibody lecanemab successfully completed a Phase III clinical trial for early AD (prodromal AD + mild AD dementia) and was granted regulatory approval and made available both in the US and Japan. Also, phase III trial of donanemab was completed successful. The J‐TRC study was initiated in Japan as a “trial ready cohort (TRC)” consisting of participants who met the eligibility criteria for participation in preclinical and prodromal AD trials. Based on such a platform, the development of DMT for AD will progress more rapidly in the future.

All of the drugs in clinical use for the treatment of Alzheimer's disease (AD), such as donepezil, which was first used in Japan in 1998, until recently were symptomatic therapies that replace deficient substances or improve neurotransmission. In contrast, the development of disease‐modifying therapies (DMTs), which are designed to suppress the neuropathological progression of AD, has been progressing rapidly in recent years.

The neuropathological changes in AD include neuronal loss in the cerebral cortex, neurofibrillary tangles (intra‐neuronal accumulation of tau protein), and senile plaques (accumulation of polymerized amyloid β peptide (Aβ) fibrils in the extracellular space). The arguments in support of the “β amyloid hypothesis,” postulating Aβ deposition as the etiology of AD, are as follows: (i) Aβ accumulation is a pathological change that occurs in the first stage during the course of AD; and (ii) Aβ accumulation is specific to AD. Most importantly, the fact that mutations in the three genes identified as pathogenic genes for autosomal dominantly inherited AD, i.e. *APP*, *PSEN1*, and *PSEN2*, altogether increased Aβ deposition, strongly support the Aβ hypothesis. There have been many arguments against the β amyloid hypothesis. The main objection is that “therapies targeting Aβ have not shown clinical efficacy in clinical trials.” Against this backdrop, the anti‐Aβ antibody drugs lecanemab and then donanemab achieved clinical endpoints in phase III trials, and lecanemab has been covered by public insurance in Japan since December 20, 2023. This article provides a brief overview of the progress in the research and development of DMT for AD.

## Development of Disease‐Modifying Drugs against Aβ

The first drugs developed to target Aβ were inhibitors of γ‐secretase, the protease that cleaves to form the C terminus of Aβ from APP. Semagacestat, a non‐competitive inhibitor of γ‐secretase developed by Eli Lilly and Company, achieved reductions in blood Aβ levels in human early phase trials, and phase 3 trials were conducted in >3000 patients with AD with mild to moderate levels of dementia, but clinical efficacy was not demonstrated and it was stopped early in 2011.^1^ The development of β‐secretase (BACE1) inhibitors was delayed until the 2010s, and the inhibitory effect of highly reducing Aβ levels in blood and cerebrospinal fluid by >50% was realized in humans. However, all clinical development was terminated due to the appearance of reversible but worsening cognitive function in several phase III trials conducted in various stages of AD.^2^

Immunotherapy against Aβ was developed in parallel with the development of secretase inhibitors. In 1999, Schenk *et al*. showed that active immunization of APP transgenic mice with Aβ peptide as a vaccine reduced Aβ deposition in brains.^3^ This led to an immediate clinical trial of vaccine therapy in humans, but the trial was terminated because of autoimmune encephalitis developed in a few percent of cases, as well as the failure to prevent progressive neurodegeneration in the early active immunization trial, despite the apparent success in amyloid clearance indicated by autopsy.^4^

However, animal experiments showed that anti‐Aβ antibodies acted as an effector in the brain, and passive immunotherapy using humanized anti‐Aβ antibody drugs has since become the mainstream therapy. In a clinical trial of the first anti‐Aβ amino‐terminal antibody, bapineuzumab, it was found that ARIA (Aβ related imaging abnormality), an adverse event resulting in transient localized brain edema, occurred in a dose‐dependent manner.^5^ ARIA is a common side effect of antibody drugs targeting aggregated Aβ, and is known as angiogenic edema associated with the removal of Aβ from the vessel wall. ARIA‐E is often silent and transient, but moderate or severe ARIA with symptoms such as headache and dizziness may require interruption or discontinuation of therapy, or even steroid treatment. Another anti‐Aβ antibody solanezumab, which preferentially clears the soluble form of Aβ,^6^ also failed in clinical trials conducted in mild to moderate AD dementia^7^ and preclinical AD,^8^ presumably because of the suboptimal target patient population and Aβ type.

Aducanumab, which was developed by Biogen in the 2010s, recognizes insoluble Aβ, and was tested in a phase 1b trial for “early AD”, the latter being a disease stage combining prodromal (mild cognitive impairment; MCI) and mild dementia stages of AD. A dose‐dependent improvement was seen in the reduction of amyloid accumulation measured by PET and cognitive/clinical functions assessed by the CDR‐sum of boxes (SB) and the Mini‐Mental Scale Examination (MMSE).^9^ EMERGE and ENGAGE phase 3 trials enrolled 1600 each of amyloid PET‐positive early AD patients and tested aducanumab, using CDR‐SB as the primary endpoint. The pre‐specified interim analysis performed in December 2018 suggested futility, which led to the termination of both trials. The final analysis showed that EMERGE demonstrated a 22% improvement in CDR‐SB progression *versus* placebo at 78 weeks, while no benefit was detected in ENGAGE, which was conducted with the same design.^10^ In June 2021, the U.S. Food and Drug Administration (FDA) granted accelerated approval to aducanumab, subject to additional post‐marketing studies, recognizing the consistently demonstrated efficacy in amyloid removal in all studies as a “surrogate” endpoint that can reasonably predict clinical benefit. However, the PMDA in Japan decided to continue the review of the drug requiring additional evidence of efficacy, and the European EMA also did not approve the drug. In the U.S., CMS decided to apply “coverage with evidence development,” which only reimburses drugs used in clinical trials and research due to lack of evidence, and the clinical use of aducanumab has not yet been achieved.

Lecanemab was originally developed by Lannfelt *et al*. in Sweden using Aβ protofibrils as immunogen, and its clinical development was conducted by Eisai. Protofibrils are an intermediate form of Aβ that are thought to be formed prior to fibrillization and exhibit neurotoxicity *in vitro*.^11^ In a Phase 2 study, in which an adaptive dose‐finding Bayesian analysis was conducted, the highest dose group of 10 mg/kg body weight, administered every 2 weeks, showed an approximately 30% improvement in cognitive decline assessed by the composite measure ADCOMS (Alzheimer's Disease Composite Score), as well as a reduction in amyloid accumulation.^12^ Phase 3 trial Clarity showed a 27.1% reduction in the progression of CDR‐SB in an 18‐month randomized trial enrolling 1795 patients with early AD, and a 5.3–7.5 month delay in progression was achieved in the active drug group *versus* placebo at the 18‐month time point.^13^ Lecanemab was approved by the U.S. FDA on July 6, 2023, and was also approved by the Japanese regulatory authorities on September 25, and was covered by the Japanese health insurance on December 20.

Donanemab is a monoclonal antibody developed by Eli Lilly and Company that specifically recognizes the pyroglutamate modification that occurs at the amino terminus of Aβ after deposition in the brain, making Aβ deposits more resistant to degradation by aminopeptidases and more specifically targeted by the therapeutic antibodies.^14^ The Phase III trial of donanemab, TRAILBRAZER‐ALZ2, demonstrated a reduction in amyloid deposition by 88‐centiloid (i.e., a standardized measure of amyloid deposition by PET imaging on a scale of 0 to 100), and a high reduction in the worsening of combined clinical endpoint iADRS (Integrated Alzheimer's Disease Rating Scale) of 22–35%.^15^ The drug is currently under review by the FDA and PMDA.

The reasons for the successful outcomes of the recent clinical trials of anti‐Aβ antibodies are: (i) the target disease stage has been advanced from the previous mild‐to‐moderate dementia stage to early AD, the latter including MCI to mild dementia stages; (ii) the application of ^18^F‐PET diagnostic reagents with a longer half life, has made it possible to perform amyloid PET in clinical trials in all patients, enabling the exclusion of non‐AD cases contaminating >20% by clinical diagnosis alone; and (iii) the establishment of the natural history and management of ARIA‐E has made it possible to use sufficient doses of the drug required for the clinical effects.

## Establishment of a Platform for Very Early DMT Trials: J‐ADNI and J‐TRC Studies

In the clinical trials of DMT for AD in the MCI or earlier stages, it is extremely difficult to accurately measure cognitive decline and to evaluate drug efficacy based on changes in the speed of progression, because symptoms are milder, and the rate of progression is slower in the early stages of the disease. In 2004, the Alzheimer's Disease Neuroimaging Initiative (ADNI) was launched in the U.S. with the aim of quantitatively establishing the natural history and precisely evaluating the effect of DMT in clinical trials.

**Fig. 1:**
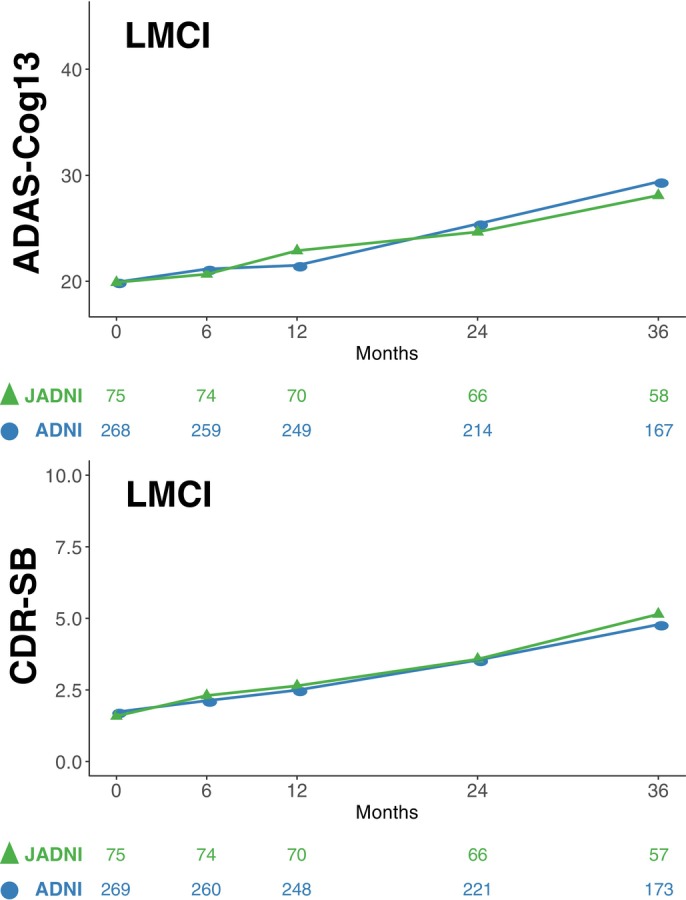
Comparison of the progression profiles of ADAS‐Cog13 and CDR‐SB in subjects of amyloid‐positive LMCI in J‐ADNI and ADNI. The mean trajectories over time in J‐ADNI (green) and ADNI (blue) are plotted as modeled with MMRM controlling for age, APOE, and education. Shaded areas in green and blue represent range of standard deviation. The numbers of subjects analyzed at each time point in J‐ADNI and ADNI are shown below each panel. Cited from reference 16.

In Japan, the J‐ADNI study was initiated in 2007, bringing together 38 clinical centers nationwide, brain imaging and biomarker researchers, industry researchers, and government officials, and ultimately completed 2–3 years of precise natural history follow‐up of a total of 537 patients, including 234 MCI cases, 149 mild AD cases, and 154 healthy elderly subjects. Approval for the J‐ADNI study protocol (UMIN000001374) was obtained from the local ethics committees or institutional review committees at the 38 participating clinical sites including the principal investigator's site (The University of Tokyo). Informed written consent was obtained from all participants at each clinical site, and the study is conformed to the provisions of the Declaration of Helsinki. A combined analysis of the results of US‐ADNI and J‐ADNI demonstrated that the progression of cognitive and clinical deterioration of “MCI due to AD,” was very comparable between the Japanese and American population, supporting the commonality in the pathophysiology and clinical progression of the early stage of AD beyond ethnicity^16^ (Fig. 1). Thus, the foundation for the harmonization in global clinical trials of AD was achieved.

**Fig. 2:**
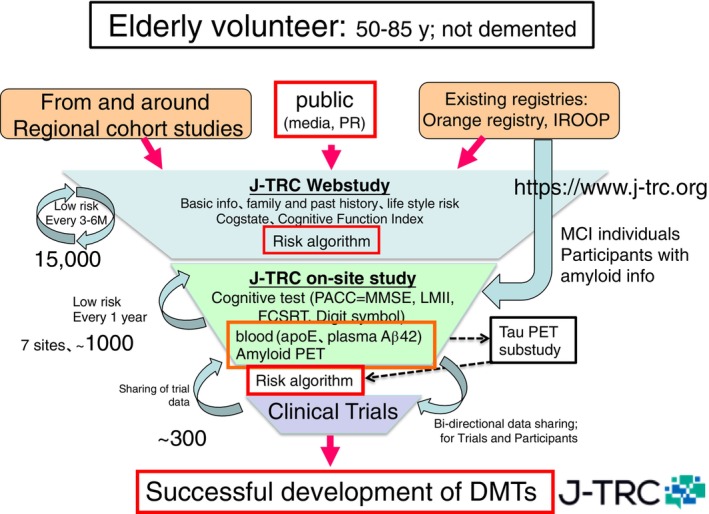
Overview of the J‐TRC study.

As the number of clinical trials for AD increases, competition for recruiting eligible subjects between academic observational studies and drug trials is increasing. Notably, prevention trials, such as the A4 trial, have been initiated for asymptomatic, preclinical stage AD, underscoring the need for an efficient system for diagnosing amyloid pathology in non‐demented, early‐stage participants. For this purpose, the construction of a “trial ready cohort”, which aims to promote both clinical trials and research, is underway worldwide. In the J‐TRC study being conducted as an AMED study, participants will be recruited and enrolled *via* the Internet in the “J‐TRC Web Study,” where digital cognitive assessment and basic information will be collected. A fraction of web participants are invited to join the “J‐TRC onsite study,” which are conducted in person at the hospital, and face‐to‐face psychological tests such as the Preclinical AD cognitive composite (PACC) will be used to sensitively quantify cognitive function changes in preclinical AD, and PET brain amyloid assessment are conducted to assess brain amyloid (Fig. 2). Approval for the J‐TRC onsite study protocol (UMIN 000038711) was obtained from the local ethics committees at the seven participating clinical sites including the principal investigator's site (The University of Tokyo). Informed written consent was obtained from all participants at each clinical site, and the study conformed to the provisions of the Declaration of Helsinki. In addition to establishing a cohort of people with preclinical AD, we are also promoting research on the predictive ability of brain amyloid using blood biomarkers such as Aβ42 and phosphorylated tau 217, which detect the brain AD pathology at the earliest stage for the future.^17^ Participants who are invited to participate in drug trials and meet eligibility requirements are provided with information about the trials upon request to support their participation; as of March 31 2024, 14,126 participants in the J‐TRC web study and 687 participants in th J‐TRC onsite study, of which more than 40 are invited to clinical trials and more than a dozen have been randomized.

## Conclusion

Antibody drugs for AD such as lecanemab was just made clinically available for people with early AD at the end of 2023. It is essential to complete post‐marketing clinical studies, especially establishing patient registries, in collaboration with industry, government, and academia to assure its safe use and to verify its efficacy in actual clinical practice. In addition, prescreening of individuals suspected of having elevated amyloid using new biomarkers such as plasma phosphorylated tau is considered essential for the successful development of AD drugs in the future, including the preclinical period. In addition to the anti‐Aβ therapies, anti‐tau therapies using anti‐tau antibodies^18^ or intrathecal administration of antisense oligonucleotides targeting tau^19^ should address the tau‐mediated neurodegeneration that occurs downstream of Aβ and is responsible for cognitive decline.

## Disclosure statement

TI has received consultancy/speaker fees from Biogen, Eisai, Eli‐Lilly and Company, and Roche/Chugai.
